# PD49 - Induced sputum versus exhaled nitric oxide for the evaluation of airway inflammation in allergic pediatric asthma patients treated with omalizumab

**DOI:** 10.1186/2045-7022-4-S1-P49

**Published:** 2014-02-28

**Authors:** Mireia Vilella, Montserrat Bosque, Juan Trujillo, Xavier Domingo, Elisabet Guijarro, Òscar Asensio, Laura Valdesoiro, Helena Larramona, Alba Maestro, Christian Domingo

**Affiliations:** 1 Corporació Sanitària Parc Taulí, Sabadell, Barcelona, Spain

## Purpose

To determine the inflammatory changes in the airways of allergic pediatric asthma patients treated with omalizumab, measured by the percentage of eosinophil in induced sputum and exhaled nitric oxide.

## Methods

From 2006 to 2012, 31 patients with asthma were treated with omalizumab (15 male -51.6%; 16 females -48,4%). Age ranged between 6 -18 years. The dose of omalizumab was calculated according to the dosing table of the company. Omalizumab was administered subcutaneously in the hospital.

## Protocol

Baseline analysis from every patient: Total IgE concentration, specific IgE against the relevant allergen, the percentage of eosinophils in a smear of induced sputum and the exhaled fraction of nitric oxide (NO). Induced sputum and NO were measured at the end of follow-up. Data are shown as mean (SEM). A Student t-test for paired data was used for comparison.

## Results

Follow-up of patients was not uniform, ranging from 2-6 years. Total IgE concentration at entry: 668.89 (117.79) IU/mL; Specific IgE against the major antigen at entry: 42.15 (7.32) IU/mL; ( 22 house dust mite; 7 alternaria; 2 cladosporium). Initial and end induced sputum: 6.26 (2.03)% vs 2.47 (0.36)% ; (p<0.05). Initial and end NO values: 19.04 (1.98) ppb vs 18.10 (2.11) ppb (p=NS).Three patients were excluded from the evaluation due to exagerated values in final NO measurement that preceded a severe exacerbation.

## Conclusions

Omalizumab allowed a statistically significant decrease in the percentage of eosinophils in induced sputum of this cohort of patients. Although very sensible, NO is a less reproducible and thus less reliable method to evaluate chronic airway inflammation in a pediatric allergic population with uncontrolled severe asthma. Induced sputum seems to be a better method to monitor chronic inflammation and thus the response to chronic omalizumab treatment while NO measurement would be more useful to monitor acute events preceding exacerbations.

